# On the Extent of Linkage Disequilibrium in the Genome of Farm Animals

**DOI:** 10.3389/fgene.2019.01304

**Published:** 2020-01-17

**Authors:** Saber Qanbari

**Affiliations:** ^1^ Leibniz Institute for Farm Animal Biology (FBN), Institute of Genetics and Biometry, Dummerstorf, Germany; ^2^ Animal Breeding and Genetics Group, Department of Animal Sciences, Center for Integrated Breeding Research, University of Göttingen, Göttingen, Germany

**Keywords:** association mapping, selection mapping, runs of homozygosity, allele frequency spectrum (AFS), haplotype block

## Abstract

Given the importance of linkage disequilibrium (LD) in gene mapping and evolutionary inferences, I characterize in this review the pattern of LD and discuss the influence of human intervention during domestication, breed establishment, and subsequent genetic improvement on shaping the genome of livestock species. To this end, I summarize data on the profile of LD based on array genotypes vs. sequencing data in cattle and chicken, two major livestock species, and compare to the human case. This comparison provides insights into the real dimension of the pairwise allelic correlation and haplo-block structuring. The dependency of LD on allelic frequency is pictured and a recently introduced metric for moderating it is outlined. In the context of the contact farm animals had with human, the impact of genetic forces including admixture, mutation, recombination rate, selection, and effective population size on LD is discussed. The review further highlights the interplay of LD with runs of homozygosity and concludes with the operational implications of the widely used association and selection mapping studies in relation to LD.

## Introduction

Linkage disequilibrium (LD) is the non-random assortment of alleles at different loci. The terms linkage and LD are often confused. As highlighted by Slatkin (2008), LD is one of those unfortunate terms that do not reveal its meaning. Indeed, LD means simply a correlation between alleles, and detecting LD does not ensure either linkage or a lack of equilibrium. This stems from the fact that mechanisms other than just physical proximity on a chromosome (linkage) such as mutation, genetic drift, and epistatic combinations might also cause (gametic phase) disequilibrium between unlinked markers. For example, admixing genetically distinct populations creates association between two loci with different allele frequencies even if they are unlinked. LD can also arise due to population stratification and cryptic relationships within a population that results in correlated allelic frequencies (reviewed in Hellwege et al., 2017).

The pattern of LD is a powerful indicator of the genetic forces shaping a population. For example, knowledge of LD helps inferring a population’s effective size (*Ne*) and past demography. Populations with smaller *Ne* experience more genetic drift than larger populations. This genetic drift causes LD between alleles at independently-segregating loci, at a rate inversely proportional to *Ne* (Waples et al., 2016). This way, an estimate of contemporary *Ne* can be concluded from LD information (Sved, 1971; Hill, 1981). On the contrary, past *Ne* is a function of LD between physically-linked loci, given that the inter-loci recombination fractions are available (Sved, 1971). Accordingly, the closely-linked loci indicate population sizes over historical past, while loosely-linked loci signify *Ne* in the immediate past (Hill, 1981, Hayes et al., 2003). Unlike the non-model species, these methods can be applied in the populations of farm animals for which the high resolution genetic maps are becoming available (Tortereau et al., 2012; Ma et al., 2015a; Petit et al., 2017).

LD between linked markers also determines the power and precision of association mapping studies,directly influencing our ability to localize genes and or loci responsible for economic traits in agriculture or inherited diseases in human (reviewed in Goddard and Hayes, 2009). Given the economic impact of domestic animals, understanding the dimension of LD enables planning and performing successful genomic breeding programs, when working towards global food security. This review aims to outline the definition of LD, summarize data on patterns of LD in the genome of farm animals, and discuss the various properties and implications that LD causes for gene mapping and evolutionary studies of livestock species.

## A Historical Glance

The concept of LD was first introduced in Jennings (1917), and its quantification (*D*) was developed by Lewontin and Kojima (1960). LD became a hot topic in the last two decades once the usefulness of LD for gene mapping became evident and genotyping of large numbers of linked single-nucleotide polymorphism (SNP) became feasible through high-throughput technologies.

The simple formulation of the commonly used LD measure *D* is the differencebetween the observed and the expected gametic haplotype frequencies comprising two loci A and Bunder linkage equilibrium (*D=P_AB_-P_A_P_B_=P_AB_P_ab_*–*P_Ab_P_a__B_*). Besides *D*, several measures of LD (for example, *D’, λ, δ, r^2^, χ* ^2^ *ρ* ^2^, among others) have been suggested (Lewontin, 1964; Bengtsson and Thomson, 1981; Hill and Weir, 1994; Terwilliger, 1995; Zhao et al., 2005; Gianola et al., 2013). The merits, comparison, and methodologies of these metrics with the utilization of biallelic or multi-allelic loci have been extensively described in the literature (e.g., Jorde, 2000; Pritchard and Przeworski, 2001; Mueller, 2004; Sved, 2009). Choosing the appropriate LD measure depends on the objective of the study, and one may perform better than another in particular situations. The two widely used measures of LD are *r^2^* and *D’. r^2^* is indicative of the correlation that a marker might have with the gene of interest and is often preferred for association studies.

## LD-Based Mapping of Genes

Identifying the genetics underlying phenotypic variation is the ultimate goal of most mapping studies. In general, there are two different, but to some extent, complementary methodologies to localize genes controlling traits. Both methodologies, outlined below, benefit from the properties of LD to accomplish the mapping task.

*Association mapping:* is the most common approach of mapping quantitative trait loci (QTLs) that takes advantage of the historic LD to connect phenotypes to genotypes. This approach detects inherited markers in the vicinity of the genetic causatives or loci controlling the complex quantitative traits. It is often performed by scanning the entire genome for significant associations between a panel of SNPs and a particular phenotype (e.g., Hayes et al., 2010). Subsequent analyses will then be required to verify the realized association independently in order to confirm that it either directly controls the trait of interest, or is linked to (in LD with) a QTL that contributes to the trait of interest.

Association analysis is based on the principle that an unbeknownst causative variant is located on a haplotype, and a marker allele in LD with the causative variant should signify (by proxy) an association with the trait of interest. Given the fact that SNPs are in LD with one another, if a common SNP affects a trait, one can probably genotype a SNP in LD with it (a “marker” SNP) and that marker will be correlated with the trait of interest.

Quantifying the extent of LD is the essential first step to determine the number of markers required to cover the entire genome in an association study with succinct power and precision. Theoretically, extensive LD reduces the number of markers required to localize an association between marker and trait but in lower resolution. In contrast, when LD promptly decays within a short distance, many markers are needed to map a gene of interest.

Although the LD-based association analysis is a powerful tool routinely applied for gene mapping, it has not been very successful for targeting genes of complex traits, especially where the causative variants are low in frequency. This is due to the fact that commercial genotyping arrays largely under-represent infrequent alleles (reviewed in Lee et al., 2014). For a detailed discussion, refer to the article by Goddard and Hayes (2009) reviewing the pros and cons of association analysis in farm animals. Here I stress the importance of LD in exploring the genetic variability underlying phenotype-genotype relationship. It is noteworthy that with the advancement of bioinformatics tools and high throughput sequencing technologies that provides the full profile of an individual’s genetic variation, it is now possible to test for the effects of every single DNA polymorphism on phenotypic variation, without requiring LD information. However, given the presence of confounding factors such as cryptic correlations in interpreting the GWAS results, LD remains useful as evidence for validation of a detected association (Bulik-Sullivan et al., 2015).

*Mapping selection:* Selection generates LD between distant loci through a “hitch-hiking” effect (Smith and Haigh, 1974), which happens when a haplotype carrying the favored allele rises in frequency so fast and drags neighboring loci to higher frequencies. Scanning the genome for long unbroken haplotypes accompanied by extensive LD can reveal past selection responding to an adaptive quality (e.g., Sabeti et al., 2002). Domestic species have been intensively selected during the recent past through domestication, breed establishment and genetic improvement and as such, have achieved tremendous phenotypic changes. Consequently, genomic regions controlling traits of economic importance are expected to exhibit footprints of selective breeding (reviewed in Qanbari and Simianer, 2014a).

## Dependency on Allelic Frequency

**Figure 1:**
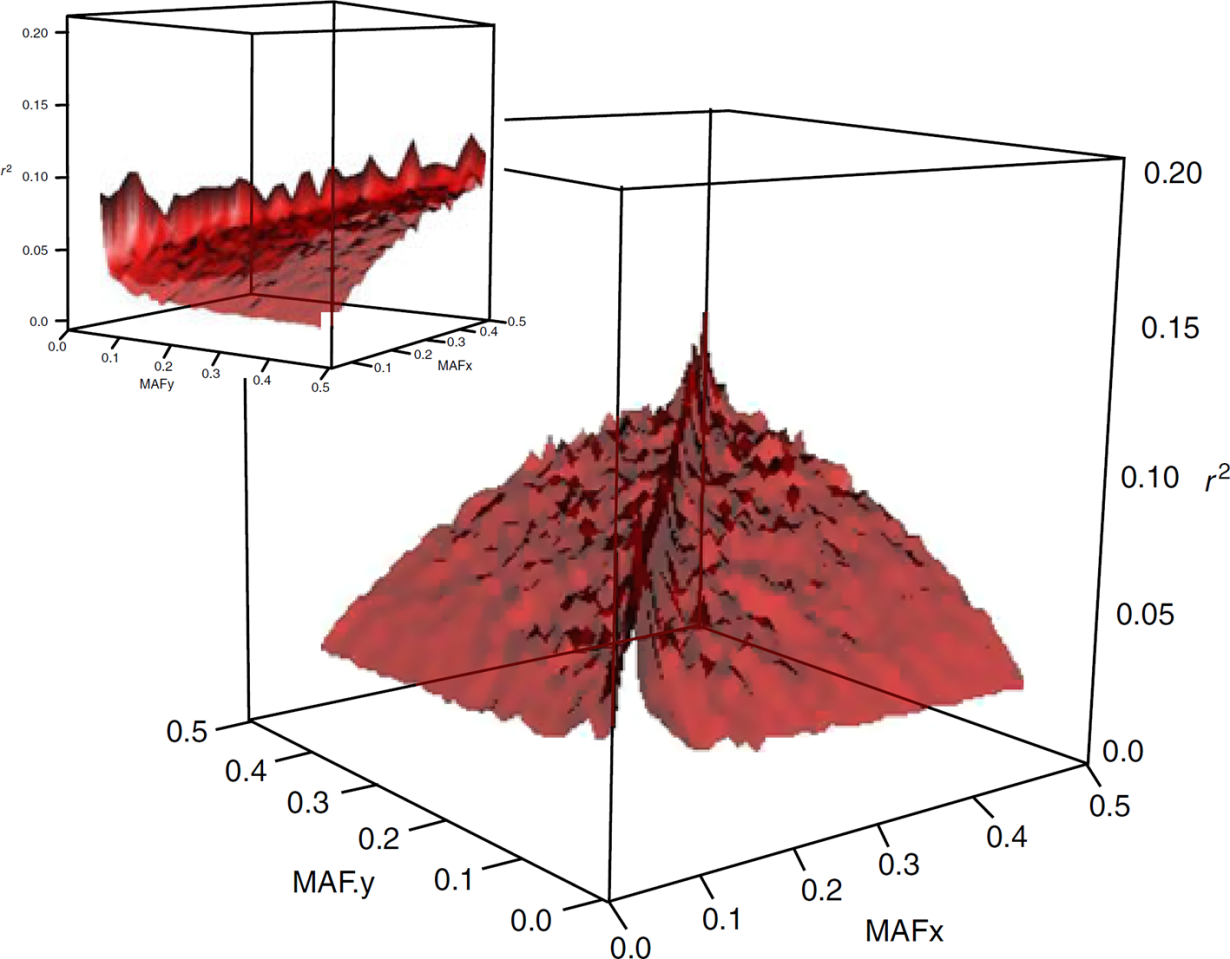
Surface plot of the dependency of LD on allelic frequency of SNP pairs. The means of *r* ^2^ are plotted for 45 bins of 0.01 allele frequency each (from Qanbari et al., 2010a).

The widely used measure of LD in animal breeding and genome-wide association mapping is *r^2^*. This metric has an allele frequency-dependent character (see **Figure 1**), as is quoted in Lewontin (1988) “*there are generally no gene frequency independent measures of association between loci”*. The dependence of *r^2^* on allele frequencies affects the outcomes and interpretations of population genetics studies in several ways. For example, there are population characteristics that are related to the estimated value of LD, such as effective population size and pattern of recombination landscapes. This implies that the estimates of effective size or recombination maps developed based on expected values of *r^2^* are frequency-dependent as well (e.g., Ober et al., 2013). Furthermore, in gene mapping studies, power to detect a causative variant using SNP markers is a function of *r^2^* between the causative variant and the marker. Thus, if a SNP marker and a causative variant have different minor allele frequencies, then the power to detect an effect at the marker can be small since high values of *r^2^* are not realized. This property of *r^2^* becomes especially more significant in human models, where the most disease-causing variants are rare and genome-wide association studies should be adapted to target these variants.

**Figure 2:**
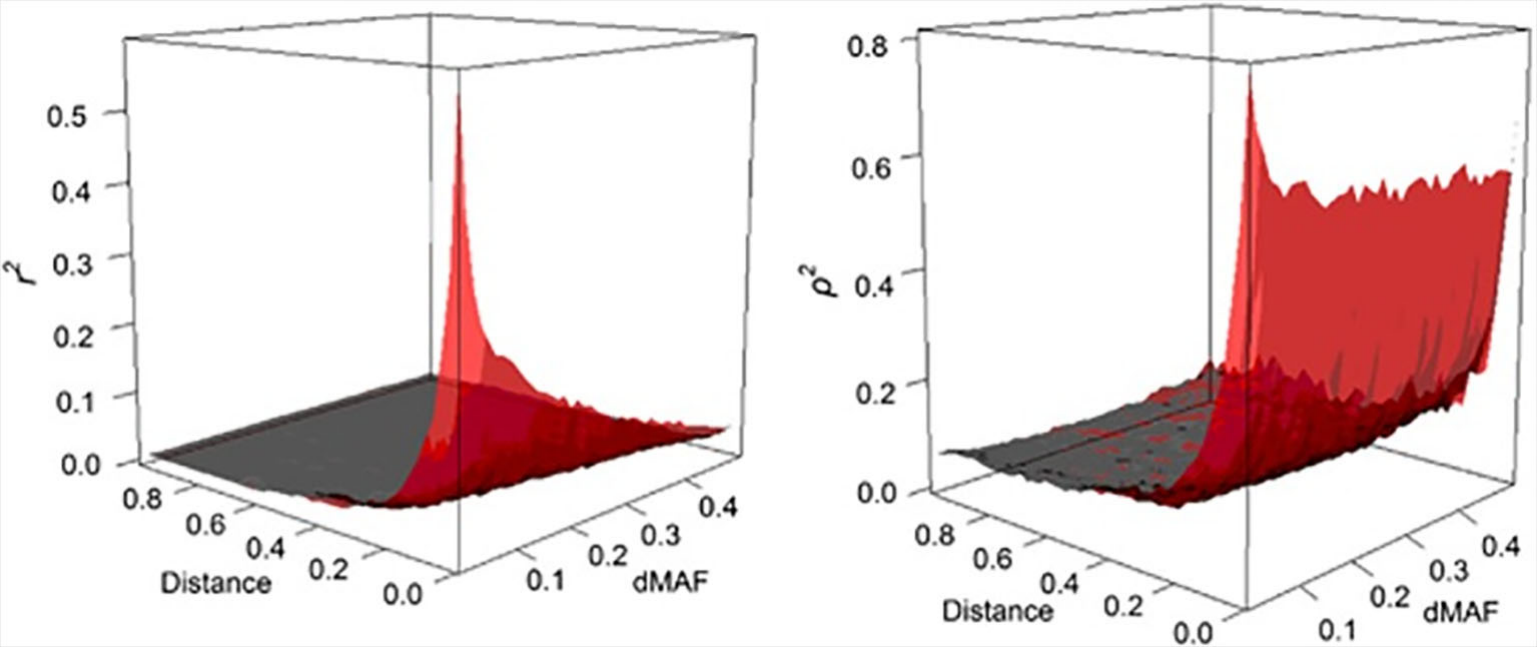
The behavior of LD as a function of inter-marker distance (Mb) and MAF interval (dMAF). The estimates of *r* ^2^ (left panel) and *ρ* ^2^ (right panel) are depicted as surface plots for SNP loci on chromosome 3 of the Italian Tuscan population in HapMap III (from Gianola et al., 2013).

Even if a frequency independent measure of LD may not exist, it would be desirable to develop one which is less affected by frequencies than *r^2^*. In a recent study (Gianola et al., 2013), we developed a new estimator of LD parameter (*ρ^2^*) based on a metric proposed by Plackett (1965) that is a tetra-choric correlation (Pearson, 1901). Plackett (1965) introduced bivariate distributions indexed by a single parameter ψ that, in the case of the 2 x 2 table, takes the form $\text{ψ }=\;\frac{P_{AA}P_{BB}}{P_{AB}P_{BA}}$. The relationship between the tetra-choric correlation and ψ is given by

$$\rho \;=\;-cos\left[\pi \frac{\sqrt{\text{ψ}}}{1+\sqrt{\text{ψ}}}\right],$$

where, *ρ* is easy to compute and much less dependent on allele frequency than *r^2^* (see **Figure 2**).

We argue that *ρ^2^* is a useful metric and potent to the further research and developments for applications in population and quantitative genetics. For instance, *ρ^2^* can facilitate comparison of levels of LD among populations that are subjected to different allelic frequencies, whereas such comparisons are distorted by the frequency-dependent nature of *r^2^*. Likewise, in the quantitative genetics context, the power analyses are formulated based on *r^2^* in association studies or genomic selection programs. For example, the sample size in indirect association studies must be increased by roughly 1/*r^2^* for detecting the causal mutation directly (Kruglyak, 1999; Pritchard and Przeworski, 2001). Similarly it is suggested that the required level of LD (*r^2^*) for genomic selection to achieve an accuracy of 0.85 for genomic breeding values has to be 0.2 (Meuwissen et al., 2001). Perhaps, similar relationships can also be developed for *ρ^2^*, which is a subject for future research.

## The Extent of LD: Genotype vs. Sequence Data

The strength of LD is of crucial importance for the genome-based analysis of evolutionary history, fine-tuning of applications like association mapping, genomic selection and selection mapping. Most of the previous studies on LD in farm animals have used panels of ascertained genotypes of different densities available by SNP genotyping arrays. The availability of population sequencing for livestock species nowadays has provided the opportunity to figure patterns of LD in unprecedented resolution. With advances in high-throughput sequencing technologies, read lengths are becoming longer, an ideal situation for estimating LD, as longer reads allow direct phasing of double heterozygotes (Maruki and Lynch, 2014).

**Figure 3:**
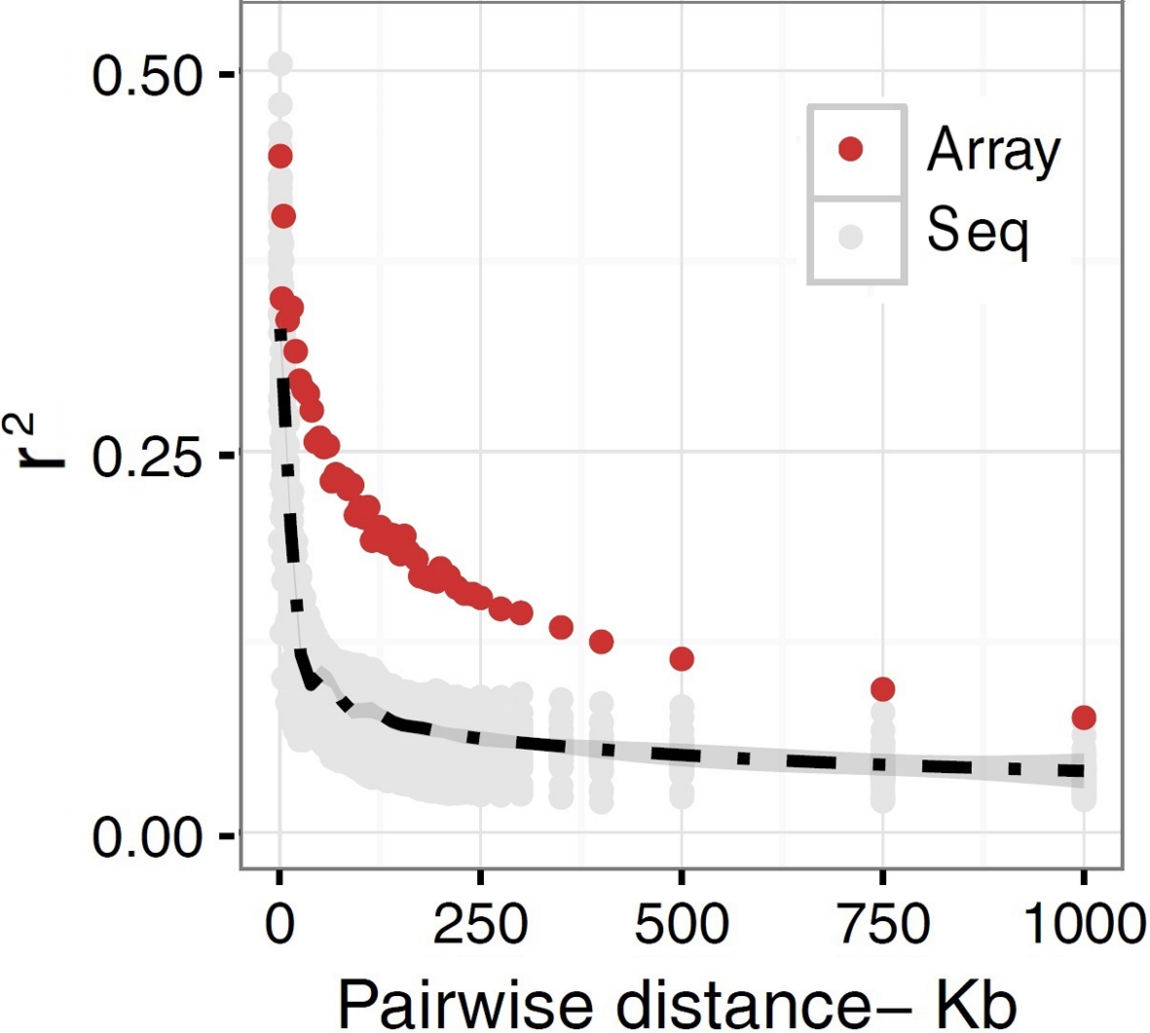
A schematic representation of decay of LD in domestic chicken. *r^2^* values are plotted as a function of pair-wise inter-marker distances based on sequence (Seq) versus SNP50K (Array) data in a population of Lohmann brown layer line. The gray dots represent sequence-based *r^2^* plotted for each chromosome separately, whereas LD based on array data was simply averaged genome-wide due to the lack of enough LD estimates in shorter distance bins. The black dashed line is fitted as mean LD in each distance bin across chromosomes. The *r^2^* values representing sequence data are estimated for sub-samples of all pairwise estimates in macrochromosomes, but include all SNP by SNP relationships in microchromosomes.

**Figure 4:**
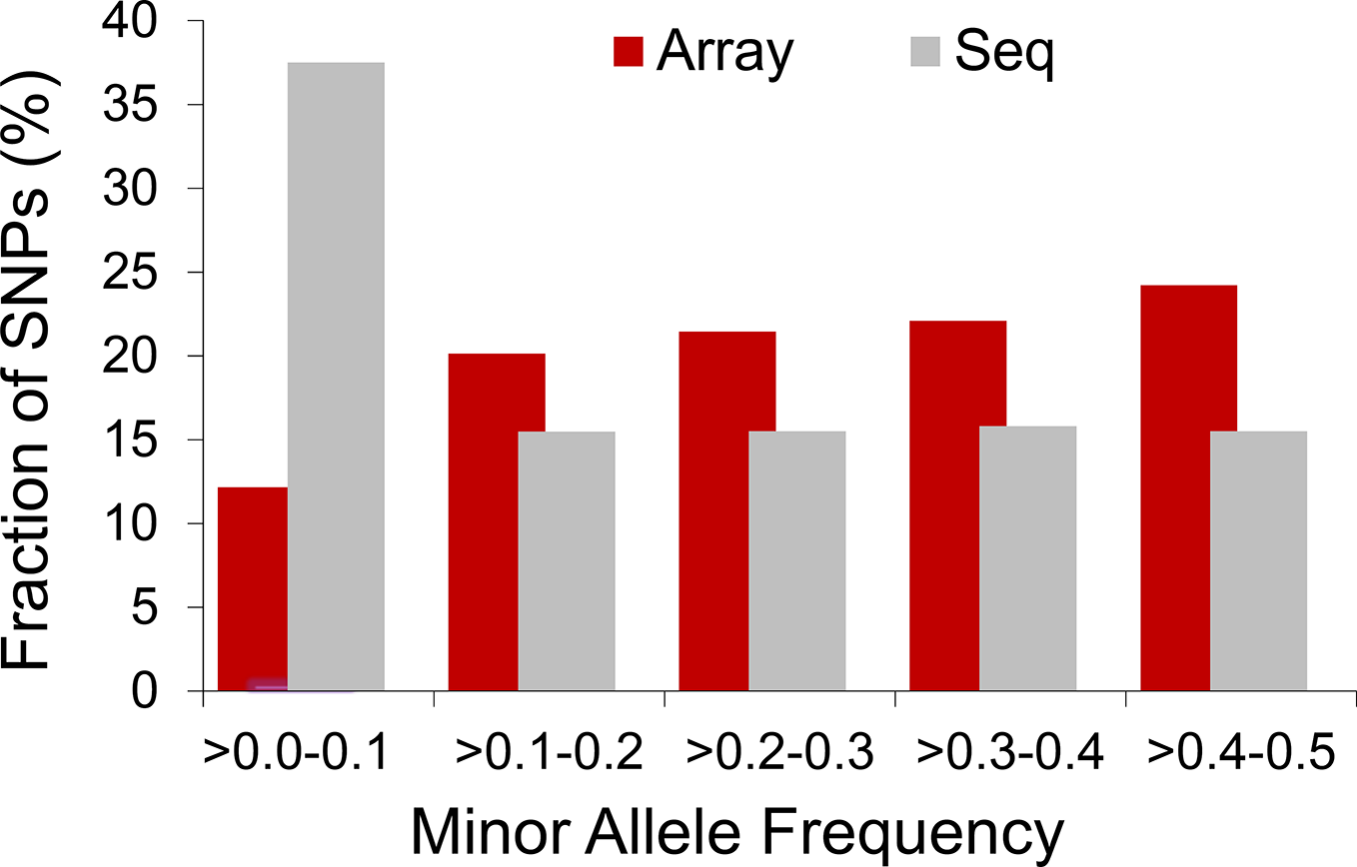
Distribution of allelic frequency in domestic chicken. Histogram compares profile of minor allele frequency between 50K array and sequence data in a population of Lohmann brown layer.

The extent to which LD decays in the genome of farm animals has been extensively studied on the basis of genotypes from SNP arrays (Porto-Neto et al., 2014; Khanyile et al., 2015; Prieur et al., 2017; Marchiori et al., 2019; Mokhber et al., 2019; Muñoz et al., 2019, among others). While genotyping arrays exhibited LD extending at several hundreds of kilobases, a denser catalog of SNPs generated from genome re-sequencing reveals LD decaying at much shorter distances (see **Figure 3**). This is attributed to the SNP profile used to measure LD. As shown in **Figure 4**, the distribution of allele frequency drawn from sequence data is a decreasing function that involves a sizable fraction of infrequent alleles. In contrast, frequency distribution in genotyping arrays is rather an increasing function, as SNPs were mainly ascertained aiming at frequent alleles and coverage of the genome during the establishment of the array (also see Fu et al., 2015 and Makina et al., 2015). Given that LD, as measured by *r^2^* depends on allele frequencies, the difference between the studies is partially due to the biased SNPs selection on the genotyping arrays. Other factors such as the influence of population sub-structuring in the sample composition or sequencing errors may also affect the allelic correlations. However, LD measures in this experiment were drawn from the identical set of samples for both array and sequence resolution and the differences between the two marker sets are too significant to be caused by sequencing errors. For further validation of this observation based on possible scenarios I refer to the experiments described in Qanbari et al. (2014b).

## LD Haplo-Blocks: Genotype vs. Sequence Data

A haplotype block is a set of closely linked markers on a chromosome with a strong LD between each other that tend to inherit together (Gabriel et al., 2002). The haplo-blocks could have been produced by interplay of several possible mechanisms, including domestication, population subdivision, founding events, selection, and recombination hotspots. These structures, when discovered, were of great practical importance for the gene mapping studies; as such, testing one SNP within each block for significant association with a trait might be sufficient to indicate association with every SNP in that block (Carlson et al., 2004). This could reduce the number of SNPs required to be tested in association studies.

**Figure 5:**
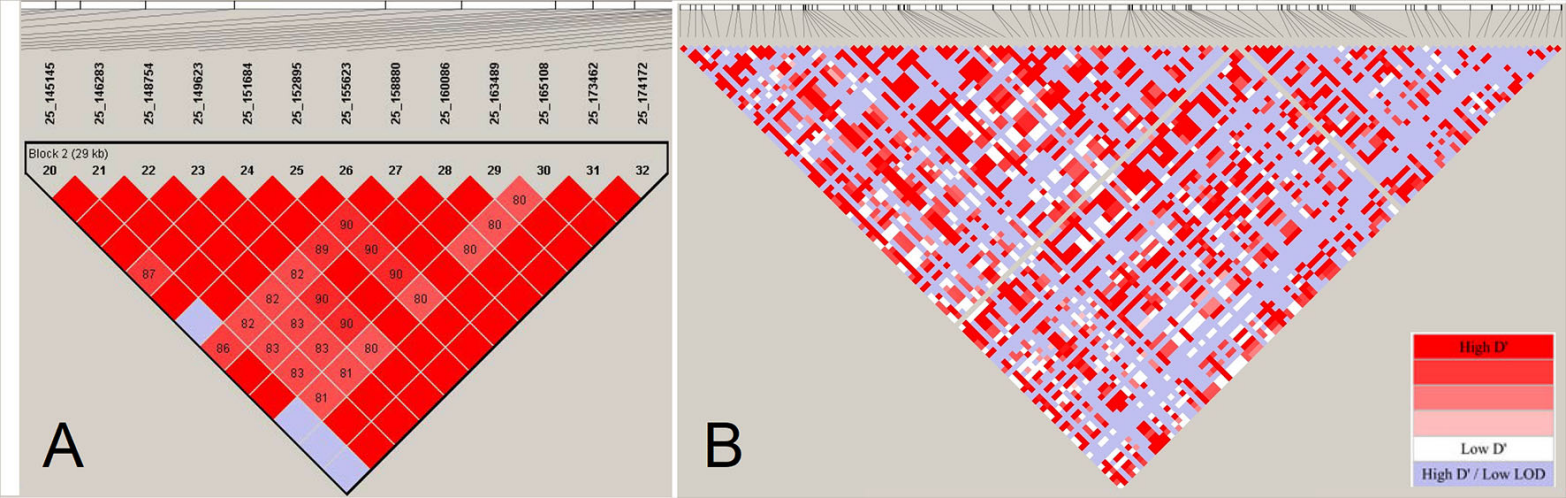
The LD-block structuring as a function of SNP density. (Panel **A**) displays a LD block of length 29 Kb based on estimates of pair-wise *D’* among 13 SNPs located on BTA25 in Fleckvieh cattle. (Panel **B**) displays LD structure in the same region in sequencing resolution consisting of 115 markers. The LD blocks are obtained using “confidence intervals” algorithm (Gabriel et al., 2002) in Haploview (Barrett et al., 2005). LD analysis has been conducted with a constant number of individuals.

Haplotype blocks have been studied in human and other farm animals. Previous studies in farm animals based on array data have reported haplo-blocks extending to several hundreds of kilobasepairs (e.g., Qanbari et al., 2010a; Qanbari et al., 2010c; Al-Mamun et al., 2015, among others). The assembly of large LD blocks appearing in array-based analyses, however, breaks into series of shorter tracts when LD is assessed by sequence data in the cattle genome (**Figure 5**). Consistent with the reduced LD profile presented in **Figure 4**, resolving large haplo-blocks in sequence resolution is a consequence of shift in allele frequency spectrum towards infrequent alleles that are under-represented in the ascertained array genotypes. This way, a sizable number of pairwise LD estimates comprising infrequent alleles become smaller so that a reduced LD profile breaks stretched LD blocks formed in the array-based experiments.

## To What Extent is LD in Farm Animals Influenced by Humans?

Addressing this question requires speculating about the possible influence of domestication,breed establishment and animal farming on genetic factors implicating LD. Principally, LD is influenced by several factors, including drift, admixture, mutation and recombination rates, selection, finite population size, population bottlenecks, or other genetic events which a population experiences (reviewed in Slatkin, 2008). For example, population admixture creates sizable LD, depending on the similarity of the allele frequency profiles in the admixed populations. LD due to crossbreeding of inbred lines is significant but, it could be small when crossing breeds have similar gene frequencies, and it erodes quickly and disappears after a limited number of generations. Mutation, due to its minor effect on changing gene frequencies, has a negligible impact on the LD in the time frame of domestication. Selection is probably a significant cause of LD, however, its effect is likely localized around specific (major) genes, and so has relatively little effect on the amount of LD averaged across the genome.

While the buildup of LD can be a result of several population genetic forces, recombination isthe only primary mechanism to break it down. The absence of recombination between sites under selection can reduce the efficiency of selection in what is known as the ‘Hill-Robertson effect’ (Hill and Robertson, 1966). It is suggested that high rates of recombination during domestication have contributed to strong selection response (reviewed in Ross-Ibarra, 2004), but remains a debate since the evidences are ambiguous and inconclusive. The most recent study found no difference in the number and distribution of recombination breakpoints between dogs and wolves suggesting that both upper and lower bounds of crossover rates may be tightly regulated (Muñoz-Fuentes et al., 2015).

**Figure 6:**
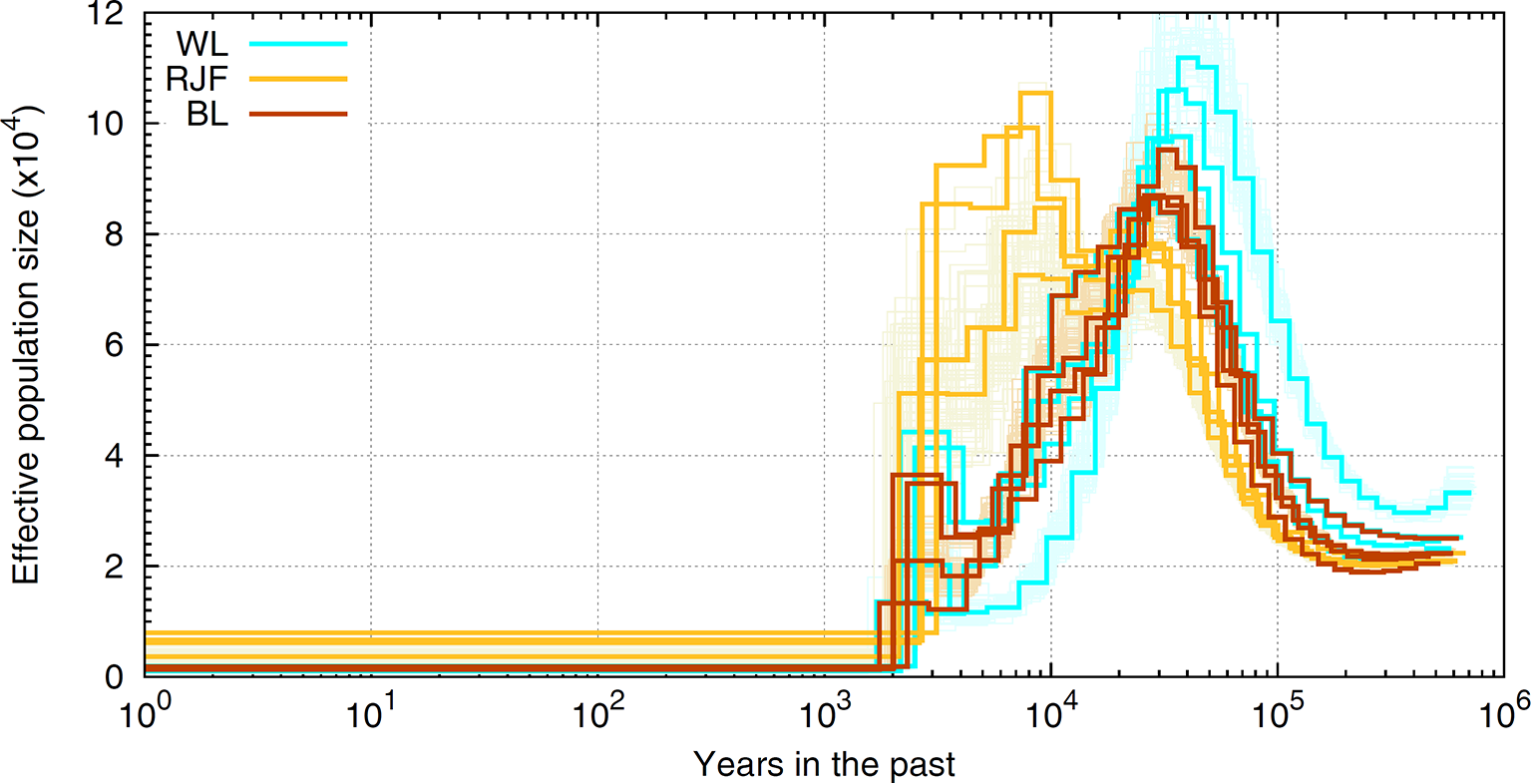
A schematic illustration of historical *Ne* in chicken. The ancestral demography is inferred in sequence resolution for RJF and white (WL) and brown (BL) layers employing the Pairwise Sequentially Markovian Coalescent [PSMC, Li and Durbin (2011)] framework. The scale on the x-axis is years in the past and the scale on the y-axis represents the historical effective population numbers. Orange (RJF), brown (BL), and cyan (WL) lines represent inferred demography for different populations with bootstraps in lighter colors. Note that inferences of bootstraps are depicted only for one sample of each population.

The finite population size is generally thought to be the leading cause of LD as effectivepopulation size has been severely eroded for most domestic species. For example, our experimentbased on sequence data suggests that chicken has experienced a drastic decline in*Ne*, evidencing a severe bottleneck most likely driven by domestication started inrecent past (see **Figure 6**). As shown, chicken hadthe largest effective population size 10,000 years ago which coincides with the generally accepted timing of chicken domestication (e.g., Xiang et al., 2014). The most recent *Ne* has dropped to a few hundred individuals and the Red Jungle Fowl (RJF) appears to have a larger population size present day in comparison to the commercial birds. A similar pattern of historical demography is observed in cattle (The Bovine HapMap Consortium, 2009). In human, the story is the opposite (The 1000 Genomes Project Consortium, 2015); improved agricultural productivity and industrialization have led to dramatic increases in population size. If LD is a result of the (current) finite population size, then the extent of LD should be many times more in livestock, as these species have *Ne* order of magnitude smaller (Leroy et al., 2013; Hall, 2016; Boitard et al., 2016) than the recent estimates reported for humans (Keinan and Clark, 2012; Browning and Browning, 2015). In reality, this is observed only for a portion of the marker pairs situated apart up to several hundreds of kilobases (Szyda et al., 2017). Instead, the observations based on full re-sequencing data revealed that the average genome-wide LD in chicken (see **Figure 4**) and cattle (Qanbari et al., 2014b) extends less than 40Kb, slightly greater than that in human populations. Since this is obtained from the full profile of polymorphisms, it represents the real strength of LD in these genomes, and far less than the extent previously reported.

Indeed, the observation of nearly comparable strength of LD in human and livestock is aconsequence of a sizable amount of polymorphism preserved in the genome of livestock. We observe millions of SNPs in the genome of cattle (e.g., Daetwyler et al., 2014) and chicken (Qanbari et al., 2019), in line with the latest updates of the genome sequencing projects in other livestock populations, including horse (Jagannathan et al., 2019), pig (Rubin et al., 2012), and sheep (Naval-Sanchez et al., 2018) that identified tens of millions SNP variants. This is comparable to the polymorphism content found in the human genome on the basis of sequencing several hundreds of individuals (The 1000 Genomes Project Consortium, 2015).

Hypothetically, the observed level of nucleotide diversity is much larger than a small population with *Ne* as low as several tens or hundreds is expected to generate or carry. This implies that chicken and cattle must have experienced much larger *Ne* in their history, which is indeed what exactly emerges from demographic inferences in these species. For example, analysis of sequence data suggests that chicken had a historical *Ne* around 25,000 at 1 million years ago that persisted for several hundreds of thousands years, before chicken population expanded starting from 50,000 to 100,000 years ago (see **Figure 6**). A somewhat similar picture of ancestral demography was also reported for the bovine genome (The Bovine HapMap Consortium 2009). Comparing the LD pattern across breeds of livestock species can reveal the influence of humans in shaping the genetic buildup. LD have been reported across breeds of cattle (Qanbari et al., 2011; Porto-Neto et al., 2014; Makina et al., 2015), sheep (Al-Mamun et al., 2015; Prieur et al., 2017), pig (Badke et al., 2012; Ai et al., 2013; Muñoz et al., 2019), buffalo (Deng et al., 2019; Mokhber et al., 2019), chicken (Khanyile et al., 2015; Hérault et al., 2018), and horse (Wade et al., 2009; McCue et al., 2012, Marchiori et al., 2019), among others. The general trend is that in local breeds or populations that experienced less intensive breeding programs, LD decays faster between distant markers than the commercial populations in which, LD extends for larger pairwise distances. For example, Holstein exhibits extensive LD than the other cattle breeds, despite having the largest contemporary population. In comparison, Indicine breeds have a lower LD than Taurine, suggestive of a larger ancestral population (e.g., Porto-Neto et al., 2014). The involvement of human in shaping genetic makeup of livestock is also evident in domestic chickens, where local breeds mostly exhibit shorter extent of LD (Khanyile et al., 2015) and among the commercials, the broilers presents faster decay of LD than layer populations (Pengelly et al., 2016; Seo et al., 2018 and Hérault et al., 2018). This is attributed to a more intensive selection scheme running over many generations during past several decades in layers resulting in a lower population haplotype diversity and a smaller *Ne*.

Further to the comparable polymorphism content, a somewhat similar pattern of allele frequency spectra (SFS) emerges in human and livestock genomes from sequence data (see Qanbari et al., 2014b and Qanbari et al., 2019). The SFS in livestock follows a decreasing trend consistent with many other organisms, including human (e.g., Nielsen et al., 2012). The distinction in livestock is that the spectra are skewed towards a larger fraction of intermediate frequencies (**Figure 4**). This is most likely stemming from an extremely small effective population size in present day livestock species and substantiates the significant under-representation of infrequent alleles in commercial breeds (e.g., see Muir et al., 2008 and Qanbari et al., 2019).

## Genome-Wide Variation in LD

Across the genome, every chromosome behaves as a unique linkage group and may experience independent demography. This is similar to the inter-species or inter-population scenarios, where it generates different profiles of LD for each unit. LD levels are also higher for sex chromosomes than autosomes because recombination on the sex chromosomes only occurs in females. Previous studies of measuring LD revealed a substantial difference among chromosomes of farm animals (e.g., Sargolzaei et al., 2008). In human models, evidence also exists for significant variation in LD across genome, between sexes and among populations (Vega et al., 2005; Baudat et al., 2010; Kong et al., 2010, among others). Besides the recombination landscape which is the primary mechanism in shaping genome-wide LD, other factors such as genetic drift, demographic forces, mutation rate, and selection play a role as well. This depicts how challenging predicting LD between two sets of polymorphism based solely on physical distance could be. The design of LD mapping experiments and placement of SNPs will, therefore, require a thorough understanding of the local interplay of these factors for precisely localizing a target locus.

## The Decay of LD in Human and Livestock

LD persists for several hundreds of kilobases at least for a portion of marker pairs in the contemporary populations of chicken and cattle (Szyda et al., 2017; Hérault et al., 2018), which causes a slightly higher LD averaged over the genome compared to human. This is primarily stemming from the “family-based LD,” a representation of the large chunks of chromosomes of founder animals segregating in the population. The consanguine parents transmit these identical-by-descent segments to the progenies and create uninterrupted stretches of homozygous genotypes, known as “run of homozygosity” (ROH), the hallmark of these autozygous segments inherited from a recent common ancestor (reviewed in Peripolli et al., 2017; Ceballos et al., 2018). The frequency, size, and distribution of ROH in the genome provide insights into the inbreeding, past demography, and selection in livestock populations (e.g., Bosse et al., 2012; Purfield et al., 2012, among others). In general, the extent of ROH islands is a function of the number of generations to the common ancestor, so that longer ROH indicate recent inbreeding, whereas ROH of older origin are generally shorter. The livestock populations involve more recent inbreeding loops through assortative mating, therefore, are expected to carry longer ROH than outbred populations like human that hold a much larger effective population size and diverse population (Gibson et al., 2006). Although a direct comparison of ROH between species in previous studies is impractical due to the lack of a gold standard in defining ROH islands, the extent to which the genome is covered by ROH tracts is expected to be higher in domestic animals relative to their wild counterparts. The long unbroken homozygosity hold in ROH islands, therefore, gives rise to an extended LD in livestock than that in human.

The unusually long ROH may also persist in outbred populations. These homozygosity islands may originate from the locally low mutation or recombination rates, or be a result of the positive selection for a favorable allele followed by the hitch-hiking of the polymorphism around the target locus (see section “Mapping selection”).

## Implications for Gene Mapping Studies

LD in sequencing resolution decays more rapidly than previously reported using array data. This enables higher resolution mapping of a trait of interest in outbred populations employing either association or selection mapping strategies. This also implies that selection mapping using haplotype-based metrics demands a panel of denser SNPs arrays to efficiently reveal patterns generated by unusually long haplotypes than medium-density arrays. The low reproducibility of the results reported in some of the first genome-wide selection studies in farm animal populations (e.g., Qanbari et al., 2010b) based on medium-density SNP arrays (~50 k SNPs) may be due to the lack of power prompted by overestimating the extent of LD demonstrated here. This is backed by our recent study in which extensive simulations were used to investigate the power of combining selection signatures detected with multiple methods under different scenarios of marker density, sample size, and selection intensity (Ma et al., 2015b). The authors showed that a reasonable power to detect selection signatures is achieved with high marker density (>1 SNP/Kb). Ultimately, uncovering older selective sweeps that carry shorter haplotypes will need sequencing resolution.

The extent of LD varies across the genomic regions, chromosomes, among populations and between species. In other words, genome-wide averaged estimates of the extent of LD may not adequately reflect LD patterns of specific regions or population groups. These observations have broader practical relevance in genomic studies of farm animals, as such the optimal number of samples and marker density in either genome-wide association or selection mapping studies may largely vary due to the extremely adverse pattern of LD within and among chromosomes. Finally, confounding population characteristics such as cryptic allelic correlations or stratification may have serious impact on pattern and structure of LD in livestock populations that need to be taken into consideration in conducting unbiased genome-wide association mapping (reviewed in Hellwege et al., 2017, also see Ma et al., 2012 and Bulik-Sullivan et al., 2015).

## LD Assessment Software Tools

Estimating LD coefficients is computationally simple and can be performed using in-house scripts when the marker density is restricted to the genotypes of SNP arrays. *r^2^* is particularly straightforward to achieve based on built-in commands as it corresponds the spearman correlation between SNPs pairs. Moreover, the standard population genetics programs, among them are Haploview (Barrett et al., 2005) and Arlequin (Excoffier et al., 2005), along with several R packages provide tools to estimate LD statistics. In sequence resolution, however, estimation LD coefficients can be computationally burdensome specifically for the mega reference panels such as genome sequencing consortiums of different livestock species. For example, a panel of 1000 genomes of a mammalian species sequenced may include over 35M shared variants, which corresponds to over 4 × 10^11^ pairwise LD coefficients within 1 Mbp windows genome-wide. A number of sophisticated programs to estimate LD statistics from sequencing data are freely available. PLINK is a widely used software toolkit for analyzing genetic data and is among the most computationally efficient tools for estimating LD (Purcell et al., 2007). VCFtools is another widely used software toolkit for manipulating and analyzing genetic data that provide utilities to estimate LD from the Variant Call Format (VCF) (Danecek et al., 2011). VCFtools works with compressed VCF files (VCF.gz) which require far less storage space than PLINK BED files; however, it can be computationally demanding for large data sets. M3VCFtools (Das et al., 2016), an extension of VCFtools uses a compact haplotype representation format called M3VCF, to estimate LD statistics. M3VCF requires far less storage than genotype formats. M3VCF toolkit provides more efficient querying and data processing and has option to convert a VCF file into M3VCf format.

## Author Contributions

The author confirms being the sole contributor of this work and has approved it for publication.

## Funding

This research is financially supported by the grants from the German Research Foundation (DFG, project ChickenSeq ID. QA55/1-1) and the Federal Ministry of Education and Research (BMBF, project CLARITY, ID. 031L0166). The funders had no role in study design, data collection and analysis, decision to publish, or preparation of the manuscript.

## Conflict of Interest

The author declares that the research was conducted in the absence of any commercial or financial relationships that could be construed as a potential conflict of interest.

## References

[B1] Ai H. Huang L. Ren J. (2013). Genetic diversity, linkage disequilibrium and selection signatures in Chinese and Western pigs revealed by genome-wide SNP markers. PloS One 8, e56001. 10.1371/journal.pone.0056001 23409110 PMC3567019

[B2] Al-Mamun H. A. Clark S. A. Kwan P. Gondro C. (2015). Genome-wide linkage disequilibrium and genetic diversity in five populations of Australian domestic sheep. Genet. Selection Evol. 47, 90. 10.1186/s12711-015-0169-6 PMC4659207 26602211

[B3] Badke Y. M. Bates R. O. Ernst C. W. Schwab C. Steibel J. P. (2012). Estimation of linkage disequilibrium in four US pig breeds. BMC Genomics 13, 24. 10.1186/1471-2164-13-24 22252454 PMC3269977

[B4] Barrett J. C. Fry B. Maller J. Daly M. J. (2005). Haploview: analysis and visualization of LD and haplotype maps. Bioinformatics 21, 263–265. 10.1093/bioinformatics/bth457 15297300

[B5] Baudat F. Buard J. Grey C. Fledel-Alon A. Ober C. Przeworski M. (2010). PRDM9 is a major determinant of meiotic recombination hotspots in humans and mice. Science 327, 836–840. 10.1126/science.1183439 20044539 PMC4295902

[B6] Bengtsson B. O. Thomson G. (1981). Measuring the strength of associations between HLA antigens and diseases. Tissue Antigens 18, 356–363. 7344182 10.1111/j.1399-0039.1981.tb01404.x

[B7] Boitard S. Rodríguez W. Jay F. Mona S. Austerlitz F. (2016). Inferring population size history from large samples of genome-wide molecular data - an approximate Bayesian computation approach. PloS Genet. 12, e1005877. 10.1371/journal.pgen.1005877 26943927 PMC4778914

[B8] Bosse M. Megens H.-J. Madsen O. Paudel Y. Frantz L. A. F. Schook L. B. (2012). Regions of Homozygosity in the porcine genome: consequence of demography and the recombination landscape. PloS Genet. 8, e1003100. 10.1371/journal.pgen.1003100 23209444 PMC3510040

[B9] Browning S. R. Browning B. L. (2015). Accurate non-parametric estimation of recent effective population size from segments of identity by descent. Am. J. Hum. Genet. 97, 404–418. 10.1016/j.ajhg.2015.07.012 26299365 PMC4564943

[B10] Bulik-Sullivan B. K. Loh P.-R. Finucane H. K. Ripke S. Yang J. Schizophrenia Working Group of the Psychiatric Genomics Consortium (2015). LD Score regression distinguishes confounding from polygenicity in genome-wide association studies. Nat. Genet. 47, 291–295. 10.1038/ng.3211 25642630 PMC4495769

[B11] Carlson C. S. Eberle M. A. Rieder M. J. Yi Q. Kruglyak L. Nickerson D. A. (2004). Selecting a maximally informative set of single-nucleotide polymorphisms for association analyses using linkage disequilibrium. Am. J. Hum. Genet. 74, 106–120. 10.1086/381000 14681826 PMC1181897

[B12] Ceballos F. C. Joshi P. K. Clark D. W. Ramsay M. Wilson J. F. (2018). Runs of homozygosity: windows into population history and trait architecture. Nat. Rev. Genet. 19, 220–234. 10.1038/nrg.2017.109 29335644

[B13] Daetwyler H. D. Capitan A. Pausch H. Stothard P. van Binsbergen R. Brøndum R. F. (2014). Whole-genome sequencing of 234 bulls facilitates mapping of monogenic and complex traits in cattle. Nat. Genet. 46, 858–865. 10.1038/ng.3034 25017103

[B14] Danecek P. Auton A. Abecasis G. Albers C. A. Banks E. DePristo M. A. (2011). The variant call format and VCFtools. Bioinformatics 27, 2156–2158. 10.1093/bioinformatics/btr330 21653522 PMC3137218

[B15] Das S. Forer L. Schönherr S. Sidore C. Locke A. E. Kwong A. (2016). Next-generation genotype imputation service and methods. Nat. Genet. 48, 1284–1287. 10.1038/ng.3656 27571263 PMC5157836

[B16] Deng T. Liang A. Liu J. Hua G. Ye T. Liu S. (2019). Genome-wide snp data revealed the extent of linkage disequilibrium, persistence of phase and effective population size in purebred and crossbred buffalo populations. front. Genet 9. 10.3389/fgene.2018.00688 PMC6332145 30671082

[B17] Excoffier L. Laval G. Schneider S. (2005). Arlequin (version 3.0): an integrated software package for population genetics data analysis. Evol. Bioinform. Online 1, 47–50. 10.1177/117693430500100003 PMC2658868 19325852

[B18] Fu W. Dekkers J. C. Lee W. R. Abasht B. (2015). Linkage disequilibrium in crossbred and pure line chickens. Genet. Sel. Evol. 47, 11. 10.1186/s12711-015-0098-4 25887184 PMC4341223

[B19] Gabriel S. B. Schaffner S. F. Nguyen H. Moore J. M. Roy J. Blumenstiel B. (2002). The structure of haplotype blocks in the human genome. Science 296, 2225–2229. 10.1126/science.1069424 12029063

[B20] Gianola D. Qanbari S. Simianer H. (2013). An evaluation of a novel estimator of linkage disequilibrium. Heredity (Edinb) 111, 275–285. 10.1038/hdy.2013.46 23921642 PMC3807269

[B21] Gibson J. Morton N. E. Collins A. (2006). Extended tracts of homozygosity in outbred human populations. Hum. Mol. Genet. 15, 789–795. 10.1093/hmg/ddi493 16436455

[B22] Goddard M. E. Hayes B. J. (2009). Mapping genes for complex traits in domestic animals and their use in breeding programmes. Nat. Rev. Genet. 10, 381–391. 10.1038/nrg2575 19448663

[B23] Hall S. J. G. (2016). Effective population sizes in cattle, sheep, horses, pigs and goats estimated from census and herdbook data. Animal 10, 1778–1785. 10.1017/S1751731116000914 27160794

[B24] Hayes B. J. Visscher P. M. McPartlan H. C. Goddard M. E. (2003). Novel multilocus measure of linkage disequilibrium to estimate past effective population size. Genome Res. 13, 635–643. 10.1101/gr.387103 12654718 PMC430161

[B25] Hayes B. J. Pryce J. Chamberlain A. J. Bowman P. J. Goddard M. E. (2010). Genetic architecture of complex traits and accuracy of genomic prediction: coat colour, milk-fat percentage, and type in Holstein cattle as contrasting model traits. PloS Genet. 6, e1001139. 10.1371/journal.pgen.1001139 20927186 PMC2944788

[B26] Hellwege J. Keaton J. Giri A. Gao X. Velez Edwards D. R. Edwards T. L. (2017). Population stratification in genetic association studies. Curr. Protoc. Hum. Genet. 95, 1.22.1–1.22.23. 10.1002/cphg.48 PMC6007879 29044472

[B27] Hérault F. Herry F. Varenne A. Burlot T. Picard–Druet D. Recoquillay J. (2018). “A linkage disequilibrium study in layers and broiler commercial chicken populations,” in Proceedings of the World Congress on Genetics Applied to Livestock Production (WCGALP)(Auckland, NZL). (2018-02-11 - 2018-02-16).

[B28] Hill W. G. Robertson A. (1966). The effect of linkage on limits to artificial selection. Genet. Res. 8, 269–294. 10.1017/S0016672300010156 5980116

[B29] Hill W. G. Weir B. S. (1994). Maximum-likelihood estimation of gene location by linkage disequilibrium. Am. J. Hum. Genet. 54, 705–714. 8128969 PMC1918089

[B30] Hill W. G. (1981). Estimation of effective population size from data on linkage disequilibrium1. Genet. Res. 38, 209–216. 10.1017/S0016672300020553

[B31] Jagannathan V. Gerber V. Rieder S. Tetens J. Thaller G. Drögemüller C. (2019). Comprehensive characterization of horse genome variation by whole-genome sequencing of 88 horses. Anim. Genet. 50, 74–77. 10.1111/age.12753 30525216

[B32] Jennings H. S. (1917). The numerical results of diverse systems of breeding, with respect to two pairs of characters, linked or independent, with special relation to the effects of linkage. Genetics 2, 97–154. 17245880 10.1093/genetics/2.2.97 PMC1193714

[B33] Jorde L. B. (2000). Linkage disequilibrium and the search for complex disease genes. Genome Res. 10, 1435–1444. 10.1101/gr.144500 11042143

[B34] Keinan A. Clark A. G. (2012). Recent explosive human population growth has resulted in an excess of rare genetic variants. Science 336, 740–743. 10.1126/science.1217283 22582263 PMC3586590

[B35] Khanyile K. S. Dzomba E. F. Muchadeyi F. C. (2015). Population genetic structure, linkage disequilibrium and effective population size of conserved and extensively raised village chicken populations of Southern Africa. Front. Genet. 6, 13. 10.3389/fgene.2015.00013 25691890 PMC4315093

[B36] Kong A. Thorleifsson G. Gudbjartsson D. F. Masson G. Sigurdsson A. Jonasdottir A. (2010). Fine-scale recombination rate differences between sexes, populations and individuals. Nature 467, 1099–1103. 10.1038/nature09525 20981099

[B37] Kruglyak L. (1999). Prospects for whole-genome linkage disequilibrium mapping of common disease genes. Nat. Genet. 22, 139–144. 10.1038/9642 10369254

[B38] Lee S. Abecasis G. R. Boehnke M. Lin X. (2014). Rare-variant association analysis: study designs and statistical tests. Am. J. Hum. Genet. 95, 5–23. 10.1016/j.ajhg.2014.06.009 24995866 PMC4085641

[B39] Leroy G. Mary-Huard T. Verrier E. Danvy S. Charvolin E. Danchin-Burge C. (2013). Methods to estimate effective population size using pedigree data: Examples in dog, sheep, cattle and horse. Genet. Selection Evol. 45, 1. 10.1186/1297-9686-45-1 PMC3599586 23281913

[B40] Lewontin R. C. Kojima K. (1960). The evolutionary dynamics of complex polymorphisms. Evolution 14, 458–472. 10.1111/j.1558-5646.1960.tb03113.x

[B41] Lewontin R. C. (1964). The interaction of selection and linkage. i. general considerations; heterotic models. Genetics 49, 49–67. 17248194 10.1093/genetics/49.1.49 PMC1210557

[B42] Lewontin R. C. (1988). On measures of gametic disequilibrium. Genet. 120 (3), 849–852. 10.1093/genetics/120.3.849 PMC1203562 3224810

[B43] Li H. Durbin R. (2011). Inference of human population history from individual whole-genome sequences. Nature 475, 493–496. 10.1038/nature10231 21753753 PMC3154645

[B44] Ma L. Wiggans G. R. Wang S. Sonstegard T. S. Yang J. Crooker B. A. (2012). Effect of sample stratification on dairy GWAS results. BMC Genomics 13, 536. 10.1186/1471-2164-13-536 23039970 PMC3496570

[B45] Ma L. O’Connell J. R. VanRaden P. M. Shen B. Padhi A. Sun C. (2015a). Cattle sex-specific recombination and genetic control from a large pedigree analysis. PloS Genet. 11, e1005387. 10.1371/journal.pgen.1005387 26540184 PMC4634960

[B46] Ma Y. Ding X. Qanbari S. Weigend S. Zhang Q. Simianer H. (2015b). Properties of different selection signature statistics and a new strategy for combining them. Heredity 115, 426–436. 10.1038/hdy.2015.42 25990878 PMC4611237

[B47] Makina S. O. Taylor J. F. van Marle-Köster E. Muchadeyi F. C. Makgahlela M. L. MacNeil M. D. (2015). Extent of linkage disequilibrium and effective population size in four South African Sanga Cattle breeds. Front. Genet. 6, 337. 10.3389/fgene.2015.00337 26648975 PMC4664654

[B48] Marchiori C. M. Pereira G. L. Maiorano A. M. Rogatto G. M. Assoni A. D. Augusto I. I. V. (2019). Linkage disequilibrium and population structure characterization in the cutting and racing lines of Quarter Horses bred in Brazil. Livestock Sci. 219, 45–51. 10.1016/j.livsci.2018.11.013

[B49] Maruki T. Lynch M. (2014). Genome-wide estimation of linkage disequilibrium from population-level high-throughput sequencing data. Genetics 197, 1303–1313. 10.1534/genetics.114.165514 24875187 PMC4125401

[B50] McCue M. E. Bannasch D. L. Petersen J. L. Gurr J. Bailey E. Binns M. M. (2012). A high density SNP array for the domestic horse and extant perissodactyla: utility for association mapping, genetic diversity, and phylogeny studies. PloS Genet. 8, e1002451. 10.1371/journal.pgen.1002451 22253606 PMC3257288

[B51] Meuwissen T. H. Hayes B. J. Goddard M. E. (2001). Prediction of total genetic value using genome-wide dense marker maps. Genetics 157, 1819–1829. 11290733 10.1093/genetics/157.4.1819 PMC1461589

[B52] Mokhber M. Shahrbabak M. M. Sadeghi M. Shahrbabak H. M. Stella A. Nicolzzi E. (2019). Study of whole genome linkage disequilibrium patterns of Iranian water buffalo breeds using the Axiom Buffalo Genotyping 90K Array. PloS One 14, e0217687. 10.1371/journal.pone.0217687 31150486 PMC6544294

[B53] Muñoz M. Bozzi R. García-Casco J. Núñez Y. Ribani A. Franci O. (2019). Genomic diversity, linkage disequilibrium and selection signatures in European local pig breeds assessed with a high density SNP chip. Sci. Rep. 9, 1–14. 10.1038/s41598-019-49830-6 31537860 PMC6753209

[B54] Muñoz-Fuentes V. Marcet-Ortega M. Alkorta-Aranburu G. Linde Forsberg C. Morrell J. M. Manzano-Piedras E. (2015). Strong artificial selection in domestic mammals did not result in an increased recombination rate. Mol. Biol. Evol. 32, 510–523. 10.1093/molbev/msu322 25414125 PMC4298180

[B55] Mueller J. C. (2004). Linkage disequilibrium for different scales and applications. Brief Bioinform. 5, 355–364. 10.1093/bib/5.4.355 15606972

[B56] Muir W. M. Wong G. K.-S. Zhang Y. Wang J. Groenen M. A. M. Crooijmans R. P. M. A. (2008). Genome-wide assessment of worldwide chicken SNP genetic diversity indicates significant absence of rare alleles in commercial breeds. PNAS 105, 17312–17317. 10.1073/pnas.0806569105 18981413 PMC2582254

[B57] Naval-Sanchez M. Nguyen Q. McWilliam S. Porto-Neto L. R. Tellam R. Vuocolo T. (2018). Sheep genome functional annotation reveals proximal regulatory elements contributed to the evolution of modern breeds. Nat. Commun. 9, 859. 10.1038/s41467-017-02809-1 29491421 PMC5830443

[B58] Nielsen R. Korneliussen T. Albrechtsen A. Li Y. Wang J. (2012). SNP calling, genotype calling, and sample allele frequency estimation from new-generation sequencing data. PloS One 7, e37558. 10.1371/journal.pone.0037558 22911679 PMC3404070

[B59] Ober U. Malinowski A. Schlather M. Simianer H. (2013). The expected linkage disequilibrium in finite populations revisited, Mannheim Available at: http://arxiv.org/pdf/1304.4856v2.pdf [Accessed June 13, 2019].

[B60] Pearson K. (1901). I. Mathematical contributions to the theory of evolution. —VII. On the correlation of characters not quantitatively measurable. Philos. Trans. R. Soc. London. Ser. A Containing Papers Math. Phys. Character 195, 1–47. 10.1098/rsta.1900.0022

[B61] Pengelly R. J. Gheyas A. A. Kuo R. Mossotto E. Seaby E. G. Burt D. W. (2016). Commercial chicken breeds exhibit highly divergent patterns of linkage disequilibrium. Heredity (Edinb) 117, 375–382. 10.1038/hdy.2016.47 27381324 PMC5061916

[B62] Peripolli E. Munari D. P. Silva M. V. G. B. Lima A. L. F. Irgang R. Baldi F. (2017). Runs of homozygosity: current knowledge and applications in livestock. Anim. Genet. 48, 255–271. 10.1111/age.12526 27910110

[B63] Petit M. Astruc J.-M. Sarry J. Drouilhet L. Fabre S. Moreno C. R. (2017). Variation in recombination rate and its genetic determinism in sheep populations. Genetics 207, 767–784. 10.1534/genetics.117.300123 28978774 PMC5629338

[B64] Plackett R. L. (1965). A class of bivariate distributions. J. Am. Stat. Assoc. 60, 516–522. 10.1080/01621459.1965.10480807

[B65] Porto-Neto L. R. Kijas J. W. Reverter A. (2014). The extent of linkage disequilibrium in beef cattle breeds using high-density SNP genotypes. Genet. Selection Evol. 46, 22. 10.1186/1297-9686-46-22 PMC4021229 24661366

[B66] Prieur V. Clarke S. M. Brito L. F. McEwan J. C. Lee M. A. Brauning R. (2017). Estimation of linkage disequilibrium and effective population size in New Zealand sheep using three different methods to create genetic maps. BMC Genet. 18, 68. 10.1186/s12863-017-0534-2 28732466 PMC5521107

[B67] Pritchard J. K. Przeworski M. (2001). Linkage disequilibrium in humans: models and data. Am. J. Hum. Genet. 69, 1–14. 10.1086/321275 11410837 PMC1226024

[B68] Purcell S. Neale B. Todd-Brown K. Thomas L. Ferreira M. A. R. Bender D. (2007). PLINK: a tool set for whole-genome association and population-based linkage analyses. Am. J. Hum. Genet. 81, 559–575. 10.1086/519795 17701901 PMC1950838

[B69] Purfield D. C. Berry D. P. McParland S. Bradley D. G. (2012). Runs of homozygosity and population history in cattle. BMC Genet. 13, 70. 10.1186/1471-2156-13-70 22888858 PMC3502433

[B70] Qanbari S. Simianer H. (2014a). Mapping signatures of positive selection in the genome of livestock. Livestock Sci. 166, 133–143. 10.1016/j.livsci.2014.05.003

[B71] Qanbari S. Pimentel E. C. G. Tetens J. Thaller G. Lichtner P. Sharifi A. R. (2010a). The pattern of linkage disequilibrium in German Holstein cattle. Anim. Genet. 41, 346–356. 10.1111/j.1365-2052.2009.02011.x 20055813

[B72] Qanbari S. Pimentel E. C. G. Tetens J. Thaller G. Lichtner P. Sharifi A. R. (2010b). A genome-wide scan for signatures of recent selection in Holstein cattle. Anim. Genet. 41, 377–389. 10.1111/j.1365-2052.2009.02016.x 20096028

[B73] Qanbari S. Hansen M. Weigend S. Preisinger R. Simianer H. (2010c). Linkage disequilibrium reveals different demographic history in egg laying chickens. BMC Genet. 11, 103. 10.1186/1471-2156-11-103 21078133 PMC3001694

[B74] Qanbari S. Gianola D. Hayes B. Schenkel F. Miller S. Moore S. (2011). Application of site and haplotype-frequency based approaches for detecting selection signatures in cattle. BMC Genomics 12, 318. 10.1186/1471-2164-12-318 21679429 PMC3146955

[B75] Qanbari S. Pausch H. Jansen S. Somel M. Strom T. M. Fries R. (2014b). Classic selective sweeps revealed by massive sequencing in cattle. PloS Genet. 10, e1004148. 10.1371/journal.pgen.1004148 24586189 PMC3937232

[B76] Qanbari S. Rubin C.-J. Maqbool K. Weigend S. Weigend A. Geibel J. (2019). Genetics of adaptation in modern chicken. PloS Genet. 15, e1007989. 10.1371/journal.pgen.1007989 31034467 PMC6508745

[B77] Ross-Ibarra J. (2004). The evolution of recombination under domestication: a test of two hypotheses. Am. Nat. 163, 105–112. 10.1086/380606 14767840

[B78] Rubin C.-J. Megens H.-J. Martinez Barrio A. Maqbool K. Sayyab S. Schwochow D. (2012). Strong signatures of selection in the domestic pig genome. Proc. Natl. Acad. Sci. U.S.A. 109, 19529–19536. 10.1073/pnas.1217149109 23151514 PMC3511700

[B79] Sabeti P. C. Reich D. E. Higgins J. M. Levine H. Z. P. Richter D. J. Schaffner S. F. (2002). Detecting recent positive selection in the human genome from haplotype structure. Nature 419, 832–837. 10.1038/nature01140 12397357

[B80] Sargolzaei M. Schenkel F. S. Jansen G. B. Schaeffer L. R. (2008). Extent of linkage disequilibrium in Holstein cattle in North America. J. Dairy Sci. 91, 2106–2117. 10.3168/jds.2007-0553 18420642

[B81] Seo D. Lee D. H. Choi N. Sudrajad P. Lee S.-H. Lee J.-H. (2018). Estimation of linkage disequilibrium and analysis of genetic diversity in Korean chicken lines. PloS One 13, e0192063. 10.1371/journal.pone.0192063 29425208 PMC5806858

[B82] Slatkin M. (2008). Linkage disequilibrium — understanding the evolutionary past and mapping the medical future. Nat. Rev. Genet. 9, 477–485. 10.1038/nrg2361 18427557 PMC5124487

[B83] Smith J. M. Haigh J. (1974). The hitch-hiking effect of a favourable gene. Genet. Res. 23, 23–35. 10.1017/S0016672300014634 4407212

[B84] Sved J. A. (1971). Linkage disequilibrium and homozygosity of chromosome segments in finite populations. Theor. Popul. Biol. 2, 125–141. 10.1016/0040-5809(71)90011-6 5170716

[B85] Sved J. A. (2009). Linkage disequilibrium and its expectation in human populations. Twin Res. Hum. Genet. 12, 35–43. 10.1375/twin.12.1.35 19210178

[B86] Szyda J. Suchocki T. Qanbari S. Liu Z. Simianer H. (2017). Assessing the degree of stratification between closely related Holstein-Friesian populations. J. Appl. Genet. 58, 521–526. 10.1007/s13353-017-0409-2 28986737 PMC5655691

[B87] Terwilliger J. D. (1995). A powerful likelihood method for the analysis of linkage disequilibrium between trait loci and one or more polymorphic marker loci. Am. J. Hum. Genet. 56, 777–787. 7887434 PMC1801166

[B88] The 1000 Genomes Project Consortium. (2015). A global reference for human genetic variation. Nature 526, 68–74. 10.1038/nature15393 26432245 PMC4750478

[B89] The Bovine HapMap Consortium Gibbs R. A. Taylor J. F. Van Tassell C. P. Barendse W. Eversole K. A. (2009). Genome-wide survey of SNP variation uncovers the genetic structure of cattle breeds. Science 324, 528–532. 10.1126/science.1167936 19390050 PMC2735092

[B90] Tortereau F. Servin B. Frantz L. Megens H.-J. Milan D. Rohrer G. (2012). A high density recombination map of the pig reveals a correlation between sex-specific recombination and GC content. BMC Genomics 13, 586. 10.1186/1471-2164-13-586 23152986 PMC3499283

[B91] Vega F. M. D. L. Isaac H. Collins A. Scafe C. R. Halldórsson B. V. Su X. (2005). The linkage disequilibrium maps of three human chromosomes across four populations reflect their demographic history and a common underlying recombination pattern. Genome Res. 15, 454–462. 10.1101/gr.3241705 15781572 PMC1074360

[B92] Wade C. M. Giulotto E. Sigurdsson S. Zoli M. Gnerre S. Imsland F. (2009). Genome sequence, comparative analysis, and population genetics of the domestic horse. Science 326, 865–867. 10.1126/science.1178158 19892987 PMC3785132

[B93] Waples R. K. Larson W. A. Waples R. S. (2016). Estimating contemporary effective population size in non-model species using linkage disequilibrium across thousands of loci. Heredity (Edinb) 117, 233–240. 10.1038/hdy.2016.60 27553452 PMC5026758

[B94] Xiang H. Gao J. Yu B. Zhou H. Cai D. Zhang Y. (2014). Early Holocene chicken domestication in northern China. Proc. Natl. Acad. Sci. U.S.A. 111, 17564–17569. 10.1073/pnas.1411882111 25422439 PMC4267363

[B95] Zhao H. Nettleton D. Soller M. Dekkers J. C. M.(2005). Evaluation of linkage disequilibrium measures betweenmulti-allelic markers as predictors of linkage disequilibrium between markers and QTL. Genet. Research 86, 77–87. 10.1017/S001667230500769X 16181525

[B96] Zhou X. Stephens M. (2012). Genome-wide efficient mixed-model analysis for association studies. Nat. Genet. 44, 821–824. 10.1038/ng.2310 22706312 PMC3386377

